# Reappraisal of PRRS Immune Control Strategies: The Way Forward

**DOI:** 10.3390/pathogens10091073

**Published:** 2021-08-24

**Authors:** Massimo Amadori, Valeria Listorti, Elisabetta Razzuoli

**Affiliations:** 1 Italian Network of Veterinary Immunology, 25125 Brescia, Italy; 2 Istituto Zooprofilattico Sperimentale del Piemonte, Liguria e Valle d’Aosta, 16129 Genoa, Italy; valeria.listorti@izsto.it (V.L.); elisabetta.razzuoli@izsto.it (E.R.)

**Keywords:** pig, PRRS, PRRS virus, immune response, disease resistance, disease control

## Abstract

The control of porcine reproductive and respiratory syndrome (PRRS) is still a major issue worldwide in the pig farming sector. Despite extensive research efforts and the practical experience gained so far, the syndrome still severely affects farmed pigs worldwide and challenges established beliefs in veterinary virology and immunology. The clinical and economic repercussions of PRRS are based on concomitant, additive features of the virus pathogenicity, host susceptibility, and the influence of environmental, microbial, and non-microbial stressors. This makes a case for integrated, multi-disciplinary research efforts, in which the three types of contributing factors are critically evaluated toward the development of successful disease control strategies. These efforts could be significantly eased by the definition of reliable markers of disease risk and virus pathogenicity. As for the host’s susceptibility to PRRSV infection and disease onset, the roles of both the innate and adaptive immune responses are still ill-defined. In particular, the overt discrepancy between passive and active immunity and the uncertain role of adaptive immunity vis-à-vis established PRRSV infection should prompt the scientific community to develop novel research schemes, in which apparently divergent and contradictory findings could be reconciled and eventually brought into a satisfactory conceptual framework.

## 1. Introduction

Porcine reproductive and respiratory syndrome (PRRS) affects farmed pigs worldwide and still causes heavy direct and indirect losses [1]. The syndrome emerged in the late 1980s, in USA, and later on in Europe, and it eventually became enzootic in most countries among farmed pigs. Late-term reproductive failure in sows with transplacental transmission of the virus, preweaning mortality of piglets, respiratory distress, anorexia, and possible cutaneous hyperemia in weaners and growers are common clinical signs of PRRS [2].

The two swine Arteriviruses sustaining PRRS (PRRSV-1 and PRRSV-2) had been previously identified as European (EU) type I, with the first strain isolated in 1991 and named “Lelystad”, and the North American (NA) type II, isolated in 1992 with the acronym ATCC VR-2332 [3]. More recently, the two viruses were classified as Betaarterivirus suid 1 and Betaarterivirus suid 2 by the International Committee on Taxonomy of Viruses (https://talk.ictvonline.org, accessed on 6 August 2021).

Whereas PRRS virus (PRRSV) infections are widely prevalent in farmed swine, the repercussions may vary, from asymptomatic, to very serious clinical courses, often depending on pig age and production phase [2]. On the whole, strong experimental and circumstantial evidence shows that the clinical outcome of PRRSV infection is the product of three components: virus virulence, host susceptibility, and environmental stressors [4]. Notably, PRRSV infection gave rise to subclinical courses over several decades, before PRRSV met the highly susceptible, lean type, rapid growth pigs reared in western Europe [4].

Extensive research has led to the development of effective diagnostic procedures, enabling timely detection of the PRRSV genome and antibody [5]. Oral fluids (OF), meat juice, and tissues obtained from the castration and tail-docking of piglets were also validated for large-scale diagnostic surveys [6].

Eradication of PRRS was shown to be possible on the basis of herd closure and strict biosafety control measures [7]; however, the underlying costs, logistics, and infrastructure needed have so far prevented the large-scale adoption of this procedure. Accordingly, the control of PRRS is usually based upon a complex of integrated control measures aimed at “stability”, i.e. a condition in which clinical signs of PRRS are absent in the breeding-herd population and PRRSV is no longer transmitted from sows to their offspring [8]. In practice, swine farms aim to co-exist with PRRSV under conditions of minimal clinical fallout and productive losses. In this respect, the prevention of PRRSV infection in suckling piglets is a foundational part of this control strategy, bearing in mind the much higher susceptibility of non-adult pigs to PRRSV [9]. 

**Figure 1:**
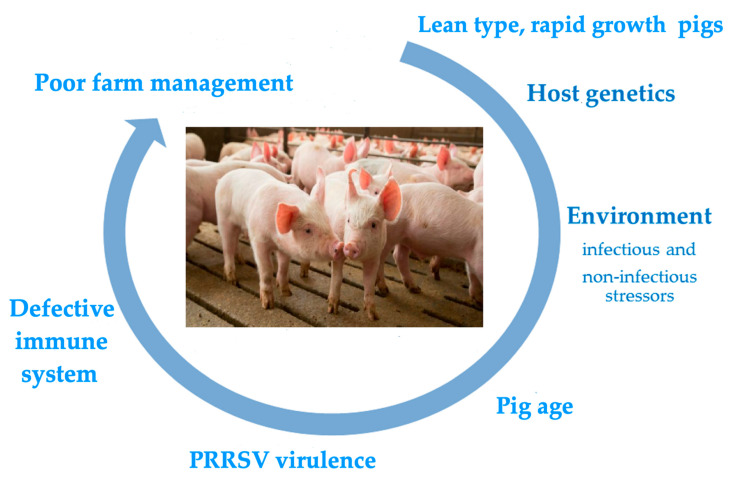
The figure depicts the main risk factors associated with serious clinical outcomes of PRRSV infection in farmed pigs. The rolling circle starts with the lean pig phenotype, which has underlain the clinical history of PRRS since the 1980s. The subsequent risk factors in the figure are not ordered on a time-related or weight basis. Pigs may actually be exposed to multiple risk factors with additive or synergistic final effects.

The main risk factors underlying serious clinical outcomes of PRRSV infection are depicted in Figure 1. All of them are dealt with in the following sections.

## 2. Biosecurity: the Foundation of Successful Disease Control Strategies

After decades of research and field experience, biosecurity is still the foundation of PRRS control on farms, as detailed, for example, in the guidelines of the American Association of Swine Veterinarians (https://www.aasv.org/aasv/PRRSV_BiosecurityManual.pdf, accessed on 3 July 2021). This implies that farm management procedures aim to reduce PRRSV infectious pressure by a proper combination of “all in—all out” protocols; parity control; limitation of cross-fostering; strict forward flow; quarantine for replacement sows and gilts; and large-scale adoption of multi-site production units, where pig groups are channeled throughout distinct production phases, giving rise to the so-called “batch management production systems (BMPS)”. These measures have been conducive to improved animal health standards compared with traditional farrow-to-finish herds, because the recirculation of pathogens and microbial infectious pressure can be more easily controlled. 

## 3. Acclimatization as the Second Pillar of Successful Disease Control on the Farm

In addition, PRRS stability demands the successful “acclimatization” of replacement gilts to the PRRSV strains circulating in the farm before the breeding period [10]. Pending a definition of reliable correlates of protection, “acclimatization” should be interpreted as a stepwise process of “adaptation” to field PRRSV strains, in which undefined innate and adaptive immune responses, the down-regulation of permissiveness to PRRSV of pig macrophages [11], and, perhaps, the “education” of macrophages to a better control of inflammatory responses by epigenetic mechanisms [12] concur to obtain a pig population that experiences PRRSV infection without serious clinical outcomes. 

## 4. Which Elements Underlie Successful Disease Control?

In retrospect, the above features related to disease control are definitely sobering. They teach us that (A) the extent of microbial infectious pressure resulting from farm biosafety profiles, and (B) the previous “education” of the immune system are both pivotal to successful disease control. 

In a wider perspective that includes the research efforts made so far, four points seem to be of paramount importance for the effective control of PRRS:

The selection of disease-resistant pig phenotypes, differing from the lean type, highly susceptible ones [13]. The high levels of oxidative stress in such pigs [14] are likely to exacerbate the inflammatory responses to infectious and non-infectious stressors and, in particular, the noxious synergism between bacterial LPS and PRRSV infection [15]. This is probably a point of some importance, since LPS can also be inhaled at high concentrations in pig herds [16], and circumstantial evidence on farm showed clinical improvement in PRRSV-infected groups after reduction of animal concentration in outdoor weaning cages. The results of extensive studies on the genetic bases of disease resistance highlighted a single nucleotide polymorphism (SNP) marker that was strongly associated with weight gain and viral load after PRRSV infection, with a possible role of the interferon-induced guanylate-binding protein gene family [17]. Moreover, editing of the CD163 gene in pig zygotes was shown to be a valuable approach for generating PRRSV-resistant animals [18]. 

Strict application of bio-safety measures toward a substantial reduction of microbial infectious pressure and chronic inflammatory responses, as well as outright “herd closure” strategies aimed at eradication [7]. 

Higher standards of animal welfare to prevent chronic stress and stress-related immunosuppression [19]. 

Active immune control, which may, in turn, include two distinct aspects: (A) Development of innate and adaptive immune responses to PRRSV [20]. (B) Reduced permissiveness of macrophages to PRRSV replication as a possible outcome of “trained immunity” [12], and/or of the inflammatory microenvironment affecting the maturation of macrophage precursors [11]. 

On the whole, the first three points are commonly accepted, and relevant measures are pursued to varying extents in different parts of the world. However, how the immune control of PRRSV takes place is a highly contentious issue, which deserves due attention and, probably, new approaches toward credible translational prospects.

## 5. PRRSV Evasion Strategies: Impact on Vaccine Performance

The dubious, inconclusive findings obtained in several studies on the immune response to PRRSV [21] are certainly related to a complex of clear decoy strategies displayed by PRRSV, as highlighted in a previous review paper of ours [4]. These decoy strategies are mainly based on the glycosylation of structural viral proteins expressing potential neutralizing epitopes [22] and non-structural viral proteins interacting with crucial check points of the innate immune response [23]. Two outcomes of the decoy strategies are of paramount importance: (A) PRRSV infection often leads to poor, late, and irregular activation of the innate and adaptive immune response [21]; and (B) PRRSV infection does not cause effective induction of cell-mediated immune responses [24,25]. Most importantly, the time-course of PRRSV viremia does not seem to be significantly correlated with the time-course of the antibody and cell-mediated immune responses [21]. As for the field data, these often show substantial discrepancies with the findings of experimental infections [26]. In addition, the performance of PRRS vaccines on the farm may be worse than expected on the basis of experimental findings [27], and concerns about the effectiveness of PRRS vaccines were repeatedly expressed in the past [28]. Interestingly, subsequent “waves” of PRRSV infection can be demonstrated in the same pigs under field conditions, as opposed to what is commonly observed in experimental trials, where re-infection of pigs with both homologous and heterologous PRRSV strains is quite difficult [29]. This makes a case for the dubious reliability of experimental PRRSV infection and vaccination studies in isolation facilities, outside of the usual complex of infectious and non-infectious stressors experienced by pigs under field conditions [26]. 

The regulation of the primary inflammatory response in macrophage precursors unable to sustain PRRSV replication is probably a further, fundamental decoy strategy of virulent PRRSV strains. A primary inflammatory response leads to the development of virus-resistant pig macrophages [11]. Accordingly, this kind of response is sustained by attenuated PRRSV strains, whereas it is inhibited to a different extent by the virulent ones [30]. Interestingly, this kind of regulation is only observed *in vitro* under non-inflammatory conditions. Instead, in leukocytes previously exposed to inflammatory stimuli, virulent PRRSV strains enhance the inflammasome reaction and the IL-1beta response [30]. This is fully in line with *in vivo* findings of PRRSV infections leading to serious clinical outcomes; these are correlated with enhanced inflammatory cytokine responses, but not with the extent of viral replication [31]. These results hint at a major upregulation of the inflammatory response following high-titered replication of PRRSV in virus-permissive pig macrophages and at a possible synergism with inflammatory stressors such as LPS [15]. Instead, virulent PRRSV strains do not exert this function in non-permissive cells; this makes sense in order to avoid any subsequent restriction of growth in differentiated pig macrophages [11]. Finally, our *in vitro* findings are in agreement with the inflammatory cytokine gene expression in the lymphoid tissues of PRRSV-infected pigs; early up-regulation of IL-1, IL-8, and IFN-gamma genes is correlated with successful virus clearance [32]. 

Interestingly, MicroRNAs (miRNAs) regulate PRRSV replication and infection. In particular, some miRNAs are reported to modulate host antiviral response; thus, miR-26a inhibits and miR-373 promotes the replication of PRRSV by up and downregulating Type I IFN genes, respectively [33,34]. In addition, miR-382-5p [35] was found to be upregulated in PRRSV infection, with a consequent inhibition of polyI:C-induced Type I IFN production after targeting heat shock protein 60.

The above findings can partly account for the unsatisfactory results of PRRS vaccines sometimes observed on farm. As stressed in our previous review paper [4], the performance of PRRS vaccines is often unpredictable on farm and cannot be easily interpreted with the current dogmas that highlight the nucleotide divergence and amino acid variability of field virus strains as the foundation of failure vs. success of vaccines [36]. Most importantly, common correlates of protection induced by vaccines have a dubious meaning in the PRRS model [37,38,39]. Furthermore, despite extensive research in this area, limited translational prospects for next generation vaccines can be foreseen in the near future. In this scenario, interesting field data have been collected for a recently licensed live attenuated vaccine, also validated for use in suckling piglets [40]. After injection into 1-day old piglets, the vaccine showed some clinical efficacy on the farm, despite the presence of maternally-derived antibodies and a concomitant infection of vaccinated piglets with a highly virulent field PRRSV strain [41]. This interesting model of the “co-existence” of wild type and attenuated PRRSV might imply the outright competition for susceptible macrophages. In this scenario, the vaccine strain could possibly successfully occupy critical macrophages niches and prevent the release of dangerous downstream inflammatory signals after wild type PRRSV infection. The better results obtained in 1-day old piglets compared with those 21-days old indirectly confirms the need for an early occupancy of the host’s macrophage compartment [41]. Needless to say, the validation of such a theory could benefit disease control in PRRS-unstable farms, characterized by extensive recirculation of virulent PRRSV strains in both sows and suckling piglets. Finally, the development of mucosal PRRS vaccines [42,43] might be pivotal for circumventing some bottlenecks in current vaccination protocols. In this respect, the possible advantages of a potent, mucosal IgA response for disease control (see Section 9) could make a case for large-scale investigations into this crucial issue. 

## 6. Virulence of PRRSV: Are There Reliable Markers?

**Figure 2:**
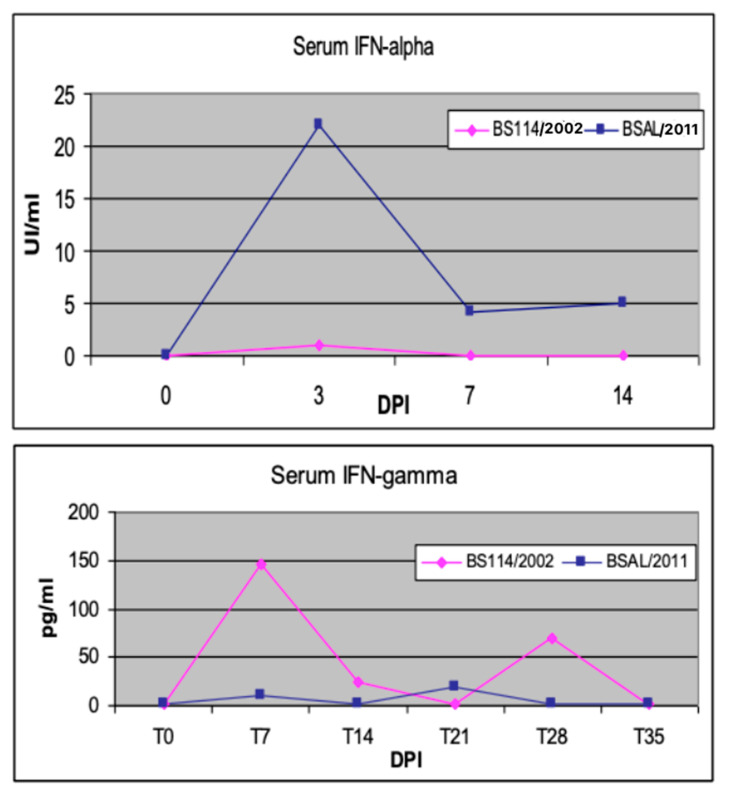
Early cytokine markers of PRRSV infection. Two groups of weaned pigs were intranasally infected with virulent BSAL/2011 and attenuated BS114/2002 PRRSV strains, respectively [45]. Blood serum samples were collected at the indicated days post infection (DPI). Swine IFN-α was measured in serum samples by a cpe-inhibition assay on MDBK cells with vesicular stomatitis virus (VSV); the test was calibrated with a preparation of porcine recombinant IFN-α1 (PBL Biomedical Laboratories, cat. 17100-1) [50]. Porcine IFN-γ was measured by ELISA with a pair of catcher and biotinylated tracer monoclonal antibodies, as previously described [27].

*In vivo*, the early interferon (IFN)-alpha response has been described as an unfavorable prognostic marker in PRRSV-infected sows [44]. Moreover, in our experience, an attenuated PRRSV strain gave rise to an early IFN-gamma response in weaners, as opposed to an early IFN-alpha response induced by a virulent PRRSV strain in the first week after infection [45] (see Figure 2). This feature should be viewed, in our opinion, in the framework of the so-called “Bad IFN-alpha response” [46], also observed, for example, in classical swine fever cases [47,48]. Diverse mechanisms (tissue damage, immunopathology, cell death) underlie the detrimental effects of inappropriate, excessive, or mistimed Type I IFN responses [46]. Conversely, effective immunomodulation in sows and piglets can be achieved by oral, low-dose IFN-alpha treatments during PRRS outbreaks [4]. The effector mechanisms of low-dose IFN-alpha were investigated in an *in vitro* model of pig tonsil cells [49]. This outlines once again the crucial roles of cytokine concentration and the regional compartment in the clinical outcome of the host’s cytokine responses and cytokine-based treatments. 

A second unfavorable marker is the late IL-10 response of PRRSV-infected pigs. In our aforementioned study [45], the plasma IL-10 response in the second week after infection was only observed in two pigs, which died a few days later. This had also been observed in a previous study of ours on breed-related disease resistance; a more serious clinical outcome of PRRSV infection in Large White pigs was correlated to a persistent, late IL-10 response [50], as opposed to the findings obtained in both Duroc and Landrace pigs. This can possibly be explained in terms of the pro-inflammatory gain of IL-10 within an established inflammatory environment, as previously shown in human models of endotoxemia [51] and Crohn’s disease [52]. How can the IL-10 response be reasonably accounted for in the PRRS scenario? In this respect, we have some reasons to postulate a central role for plasmacytoid dendritic cells (pDCs). In pigs as well, these cells can release huge amounts of IFN-alpha following exposure to viral agents [53], including many PRRSV strains [54]. In turn, IFN-alpha can induce the high-titered release of IL-10 in LPS-stimulated monocytes and CD4+ T cells [55]. This is probably the mechanism underlying the IL-10 response *in vitro* of swine PBMC to some PRRSV strains [30,56]. This would also be consistent with the above-mentioned, postulated role of IL-10 as a virulence marker *in vivo*. Finally, IFN-alpha and IL-10 can promote the differentiation of Type I T regulatory (T reg) cells [57]. The differentiation of T reg cells is promoted by PRRSV-infected dendritic cells, and this was implicated as a possible cause of virus-driven immunosuppression [58]. This makes a case for new studies into a possible PRRSV/pDCs/IFN alpha/IL-10 loop from *ex vivo* samples of PRRSV-infected pigs. 

A recent study [59] suggested investigating the Wnt/β-catenin signaling pathway to obtain information about virus–host interactions and virus pathogenicity. Indeed PRRSV-infected cells show accumulation of β-catenin in the nucleus; the activation of the Wnt pathway could be caused by PRRSV nonstructural proteins (Nsps) 1α, 1β, 3, 4, 7, 10, and 12. This activation tends to inhibit PRRSV replication by enhancing the NF-κB-dependent innate immune response. Accordingly, PRRSV strains that inhibit the Wnt pathway are probably more pathogenic than those that exalt the same pathway [60].

## 7. What Can We Learn from Other Models of Immune Response to Arterivirus Infection? 

The members of the genus Arterivirus include equine arteritis virus (EAV), the lactate dehydrogenase-elevating virus (LDV) of mice, simian hemorrhagic fever virus (SHFV), and PRRSV. The infection is strictly species-specific; however, these viruses share many common properties such as the ability to establish persistent infections [61]. 

The dubious results of the studies on the adaptive immune response to PRRSV are consistent with similar findings about other animal Arteriviruses. As mentioned in our previous review paper [4], in the murine Arterivirus model there is no difference in terms of viremia between immunocompetent and tolerant mice [62], which substantially detracts from an important role of the adaptive immune response. 

Furthermore, neutralizing antibodies for EAV, PRRSV (both North American and European), and LDV are often specific to the GP5 major envelope glycoprotein encoded by ORF5. The GP5 proteins of these viruses are similar in size (199–255 amino acids) and location, and major neutralization determinants are included in the N-terminal ectodomain. Similarly to PRRSV, EAV can maintain a persistent infection in male reproductive tissues without clinical manifestations. This guarantees shedding in semen and sexual transmission despite the onset of neutralizing antibody responses [63]. 

The cell-mediated immune response (CMI) to equine arteritis virus has not been well characterized. It is known that specific CTL precursors may persist for at least 1 year after infection, and CD8+ T-cell-mediated cytotoxicity is virus strain-specific and genetically restricted. In LDV infection, specific CD4+ and CD8+ T-cell responses do not lead to virus clearance [64].

## 8. Is There an Effective Antibody Response to PRRSV? 

The role of the antibody response to PRRS virus is highly contentious. The issue of target proteins of the neutralizing antibody (NA) and relevant neutralizing epitopes has stimulated several studies. Thus, neutralizing GP5-specific monoclonal Abs have been isolated from the sera of hyperimmune sows [65], confirming the presence of neutralizing epitopes on GP5 glycoprotein [66,67]. Neutralizing epitopes have also been observed on the minor surface glycoproteins GP2, GP3, and GP4 [68]. The isolation of specific monoclonal Abs against these proteins would likely uncover additional neutralizing or potentially broadly neutralizing antibodies [65]. It has been observed that the nsp2 protein also contributes to the neutralizing activity of the structural proteins GP5-M in vitro, suggesting that there is at least one neutralizing epitope in nsp2, or in the spatial structure formed by nsp2 and the structural proteins together [69]. The targeting of neutralizing antibodies is a relevant field of research. In this regard, an experimental system to enable the isolation of PRSSV-specific monoclonal Abs has been recently developed [70]. An important step forward could be the administration of neutralizing monoclonal Abs using mRNA technology [71]. This technology has been studied toward a possible mRNA-based therapy in swine [72,73], and a mRNA-encoded antibody has been successfully investigated as a means of protecting against HIV and rabies virus infections [74,75]. In the future, this could be a useful tool for the possible treatment of infectious animal diseases, including PRRS, where the virus has developed outright decoy strategies to prevent Ab binding and neutralization [22].

As for the role of antibodies in protective immunity, this was advocated in studies on the passive immunization of sows, even though the immune serum did not prevent PRRSV replication in target tissues in young weaned pigs nor transmission to susceptible animals [76,77]. These results in sows were confirmed in another study on intraperitoneal administration of purified, neutralizing, PRRSV-specific antibody in 3-week old piglets; this implied a reduction of viremia levels after challenge infection with both homologous and heterologous PRRSV strains [78]. However, no difference in terms of clinical course and average daily weight gain was observed between the antibody-treated and control pigs [78]. Moreover, a commercial inactivated vaccine was shown to evoke a vigorous post-challenge anamnestic NA response and a lack of protection [37], and the long-term persistence of PRRSV viremia may be possible in the presence of neutralizing antibodies [39]. Finally, high-titered antibody responses to PRRSV can even be correlated with a worse clinical outcome of PRRS [31], as also reported following administration of DNA-based PRRS vaccines and challenge infection [38]. This is in agreement with previous studies showing a role of some PRRSV-specific IgG antibodies in antibody-dependent enhancement (ADE) of infection [79]. On the whole, some protection is afforded by antibodies following passive immunization, but there is little, if any, evidence of Ab-mediated protection following vaccination and PRRSV infection. This is a point of major importance that is difficult to reconcile with current concepts about the adaptive immune response, and which possibly demands a new, relevant conceptual framework. Interestingly, a discrepancy between passive and active immunity was also evidenced in the case of African swine fever virus (ASFV) infection. Whereas the correlates of protection to an established ASFV infection are still ill-defined [80], a study suggested that colostrum/milk from sows that survived ASFV infection had a protective effect in their offspring, in terms of reduced viremia and clinical signs in response to ASFV challenge [81]. Please notice, however, that absorption of colostrum and milk from sows also implies the passage of leukocytes, including T lymphocytes, and the transfer of relevant effector functions [82]. Thus, protection cannot be unambiguously referred to immunoglobulins in the case of colostral immunity. 

Beyond the aspects of cell-mediated immunity, the passive transfer of immunoglobulins brings about Ig-driven immunoregulatory control actions other than the provision of specific antibodies to viral agents. These were clearly evidenced in human transfused patients: immune sera would lead to a control of the inflammatory response based on monomeric IgA. As opposed to IgA immunocomplexes, free monomeric IgAs underlie a potent control circuit based on their interaction with Fcα RI (CD89) on myeloid cells; after the contact, CD89 binds to the ITAM sequence of the Fc gamma chain subunit and recruits a tyrosine phosphatase, without activating any downstream kynases [83]. The outcome is very clear: free monomeric IgAs dampen the response of activated granulocytes and prevent complement deposition; they also inhibit Th17 and IFN-gamma responses, while promoting Treg development [84]. “Switch-off” of the Th17 response has been repeatedly observed in human patients after endovenous administration of Ig [85]. 

On the whole, all the experiments based on passive transfer of immune serum/plasma should be critically evaluated because of the side effects of components with the potential to affect the “cytokine storm” during viral diseases. 

## 9. The IgA Puzzle

As opposed to the aforementioned, contradictory findings about the serum antibody response to PRRSV, the peak IgA mucosal antibody response is clearly associated, in our experience, to a block of PRRSV shedding in OF [86]. Can this finding be reconciled with the above properties of IgA? The answer is reasonably affirmative. Mucosal IgA is mostly dimeric. Free dimeric IgA has limited affinity for CD89 [83]. On the contrary, IgA-virus immunocomplexes have a high affinity for CD89 and give rise to full activation of ITAM in the adjoining gamma-chain subunit of Fc gamma receptor, followed by a strong inflammatory response of myeloid cells [83]. Most importantly, the inflammatory phenotype of macrophages is highly correlated with non-permissiveness for PRRSV replication [11]. This could reasonably be the added value of the mucosal IgA response to PRRSV, possibly more important than the direct antiviral effector functions of IgA. 

Therefore, the presence of IgA-PRRSV complexes is conducive to the control of PRRSV infection, whereas IgG-PRRSV complexes may even be associated with ADE [79]. Accordingly, OF samples with moderate IgA Ab titers for PRRSV cause a yield reduction of PRRSV replication in MO cultures, as opposed to OF samples with little, if any, IgA antibodies to PRRSV [87]. Interestingly, a balance in OF between the IgG and IgA antibody response to PRRSV was not observed over several weeks in PRRS-unstable herds experiencing overt clinical cases; once a balance is observed, virus shedding in OF comes to an end, despite the ongoing viremia [86]. This makes the case for an important role of antiviral Ig isotypes and their molar ratios in shaping the effective control of PRRSV infection in tissues. In this respect, the direct antiviral, neutralizing effects of IgA might even be exerted in the intracellular compartment, in agreement with the Influenza virus model [88]. 

Moreover, local T cell responses, measured in the lungs, bronchioalveolar lavages, and bronchial lymph nodes, are induced faster than systemic responses and are maintained at significantly higher levels, even after virus clearance in experimentally infected pigs, substantiating a possible role of local immune responses in the clearance of PRRSV from pigs [89]. 

## 10. Cell-Mediated Immunity to PRRSV: What Are We Measuring?

Studies on PRRSV-specific, cell-mediated immunity (CMI) have provided conflicting results. Three points are of paramount importance: (A) There is uncertainty as to whether PRRSV proteins are effectively presented to the immune system [24]. (B) There is no correlation between the time-course of adaptive immunity and the resolution of viremia in PRRSV-infected pigs [21]. (C) PRRSV infection causes a strong inhibition of Ag presentation in macrophages [90] and can give rise to a potent induction of suppressor T reg cells [58]. Moreover, transient depletion of CD8+ T cells does not exacerbate PRRSV infection, and no effect on the ability to clear the virus has been highlighted [91]. Bearing in mind these fundamental findings, the observed responses should be viewed with some caution. In swine infected with PRRSV, CMI responses are characterized by IFN gamma-secreting, CD8+ and CD4+/CD8+ double-positive T cells, detectable 2–3 weeks post-infection and showing an erratic behavior [92]. 

Accordingly, several groups have reported the demonstration of PRRSV-specific, IFN-gamma secreting cells (SCs) with ELISPOT assays using tissue culture-adapted PRRSV as a stimulating agent [39,93]. However, the correlation between the extent of this response and the protection of sows is definitely ill-defined [94]. As a matter of fact, this approach may be affected by concomitant, non-specific IFN-gamma responses to the stress antigens carried by the established cell lines in which PRRSV is grown [95]. This is the reason why we have always employed a control Ag, i.e., a cryo-lysate of uninfected cells processed exactly as the raw PRRSV, and subtracted this response from the virus-specific one in both ELISPOT and whole blood cytokine release assays [95]; in this way, the PRRSV-specific IFN-gamma response proved to be transient and low-titered in our experience, following field PRRSV infection [86]. Interestingly, such a response is probably absent in PRRS-unstable herds, except in suckling piglets [86] as possible activity of maternally-derived immune cells, in agreement with a previous study [82]. 

## 11. Natural Killer (NK) Cells: A Missing Link?

The possible role of NK cells in PRRSV infection was highlighted in our previous review paper [4]. Why could NK cells actually play a crucial role? First, NK cells can rapidly recognize virus-induced changes in virus-infected cells in terms of both missing self and induced self [96]. Most importantly, they can mount an early IFN-gamma response, which can suppress TLR-mediated IL-10 production and inhibit expression of CD163 in macrophages [97], thus regulating the susceptibility of cells to PRRSV infection [97]. In this respect, we can surmise that the crucial role of the IFN-gamma response *in vivo* is probably related to a block of PRRSV replication in macrophages. As for NK cells, the early IFN-gamma response to PRRSV infection [98] and the infiltration of CD3−, CD8+, allegedly NK cells into the PRRSV-positive endometrium [99] are in line with an important role for these cells, which undoubtedly deserves further studies *in vitro* and *in vivo*. On the other hand, there is also evidence of impaired NK cell cytotoxicity following PRRSV infection [100], which might bear on the potential role of NK cells in virus clearance. 

## 12. Theoretical Strength and Weakness of the “Trained Immunity” Model in PRRS

The uncertain role of the adaptive immune response to PRRSV should lead to a major reappraisal of innate immunity. In particular, within the innate immune response, the epigenetic features underlying “trained immunity” [12] could be of crucial importance; they might even lead to a convincing explanation of some contradictory findings in experimental studies and field trials. Some questions do need an answer. For instance, why is vaccine-induced immunity under experimental and field conditions so variable and sometimes disappointing [27,28]? How can a vaccine sometimes induce better protection to a heterologous strain [36] or be effective against a different Arterivirus (other PRRSV genotype) [101]? Which mechanisms underlie the “acclimatization” of gilts and sows [86]? Is it a matter of adaptive immune response or a sort of “habituation” of macrophages to field PRRSV strains? 

Needless to say, one could postulate that both vaccines and exposure to field PRRSV strains induce major epigenetic changes in the innate immunity genes of myeloid cells underlying the aforementioned status of “trained immunity”. This is also in line with the possible major role of NK cells, whose activity can be also prone to crucial epigenetic regulation [102]. In this respect, there is evidence of increased cytotoxicity against NK target cells after *in vitro* re-stimulation of PBMC from convalescent pigs with PRRS virus [103]. Interestingly, after infection with a highly virulent PRRSV strain, the DNA from PBMC showed a strong hypomethylation, as opposed to the control pigs and pigs infected with an attenuated PRRSV strain (Amadori M:, unpublished results). This makes the case for new studies into the status of chromatin in the promoter regions of genes involved in the innate immune response, with careful matching between macrophages of PRRS-naive, vaccinated, and infected pigs. On the whole, the effects of such mechanisms could be two-fold: (A) They could underlie the non-permissiveness of pig macrophages to PRRSV infection after shifting to a M1-like phenotype [11]; (B) They could prevent a further major amplification of the inflammatory cytokine response, associated with adverse clinical outcomes of PRRSV infection [31]. Although such a theory is in agreement with several experimental findings, it has to be demonstrated on a convincing experimental basis. This is potentially in conflict with the paucity of validated models of epigenetic rearrangements of innate immunity genes in pigs. 

Last, but not least, this area of investigation might be relevant to the crucial link between cell metabolism and PRRSV replication. PRRSV infection is suppressed when *de novo* synthesis of fatty acids is inhibited [104]. Interestingly, free fatty acids are low-affinity ligands of PPAR-γ, which dampens the inflammatory response of macrophages [105]. In this respect, “trained immunity” implies a shift of cellular metabolism to anaerobic glycolysis [12], which affects the efficiency of *de novo* synthesis of fatty acids. As a matter of fact, the induction of M1 macrophages with inflammatory stimuli leads to a profound shift of energy metabolism without *de novo* fatty acids synthesis [106], in a scenario of little, if any, susceptibility to PRRSV [11]. 

## 13. Crucial Areas of Investigation into the PRRSV–Host Relationship

Our studies *in vitro* [30] outlined the importance of the inflammasome response and IL-1beta production during PRRSV infection. In LPS-treated macrophages, PRRSV can enhance the inflammasome response with the small envelope protein E, giving rise to an increased release of IL-1beta [107]. Such a regulatory action is counteracted by nsp11 [108]. This is in agreement with the observed kinetics of the inflammasome reaction during PRRSV infection, which shows rapid induction and decay [108]. This highlights the importance of further investigating such a crucial regulation by sequencing protein E and nsp 11 in reputedly virulent and attenuated PRRSV strains. In addition, deletions of nsp 2 could also play a role. These were observed, for example, in virulent Chinese PRRSV strains [109]. Interestingly, some nsp2 deletions were shown to reduce the expression of IL-1 beta [100]. This might be relevant to a potentially important decoy strategy of PRRSV, i.e., the inhibition of the primary inflammatory response in macrophage precursors, in order to prevent the differentiation of PRRSV-resistant, mature pig macrophages [30].

Concerning IL-10 and its role in PRRSV pathogenicity, it should be stressed that induction of IL-10 is correlated with the expression of PRRSV protein N [100]. Therefore, it could be worth comparing the N gene sequences of strains inducing or not inducing IL-10 *in vitro*, to define further virulence markers. 

## 14. Translational Prospects of Current Studies: Some Open Issues

The above sections outlined the scope of reappraised PRRS control strategies. The contributions should be channeled into five main areas of discussion, underlying the major issues with crucial translational repercussions. The five areas can be stated as follows:

Farm management. What is pivotal to PRRS control on the farm? Can we currently define “minimum requirements” of biosafety toward effective PRRS control? Can eradication sometimes be cost-effective and sustainable in the long term? Which monitoring actions should be implemented toward effective surveillance? What about the roles of clinical inspection, post mortem examination, and laboratory investigations? Can oral fluids and other unconventional organ specimens fully replace blood for PRRS surveillance? [110]

Genetic selection. Can we postulate reasonable translational applications deriving from pig genetic studies? Have we fully defined a set of critical genes underlying disease resistance? Does resistance entail clinical or virological protection, or both? What role is played by the very high, constitutive oxidative stress in rapid growth, lean type pigs [14]? Can we breed against this trait? What about the autochthonous pig breeds that usually experience PRRSV infections without serious clinical outcomes? Can they be a model for fundamental resistance traits lost during genetic selection? On the whole, such genetic markers are still ill-defined or unlikely to provide translational prospects in the near future. Nevertheless, this is still a priority for both applied and fundamental research, with a huge potential impact on pig breeding systems. 

Animal welfare. Which environmental conditions are more strictly related to PRRSV infection prevalence and serious clinical outcomes thereof? How can one effectively prevent chronic stress and stress-related immunosuppression? Which laboratory procedures can best depict immunosuppression in pigs? 

Immune control. Can we improve the performance of PRRS vaccines on farm? In the case of a positive answer, which vaccines are best suited for disease control? Can we think of effective, adaptive immune responses to PRRSV, or do we also need to think of “habituation” to the virus by means of “trained immunity” mechanisms? Is the “acclimatization” of sows and gilts based on a peculiar form of “trained immunity”? On the whole, as reported in a recent study of ours [87], the emerging picture in the PRRS model outlines unusual effector roles of adaptive immunity: both IgA Ab and cell-mediated immune responses (IFN-γ SCs) can coincide for a major modulation of macrophage permissiveness to PRRSV, as a foundation of disease control. Finally, immunomodulation by oral, low-dose IFN-alpha treatments has shown some efficacy vis-à-vis field PRRS outbreaks [4]; the peculiarities of this approach have been evaluated in a recent review paper of ours [111].

Markers of risk. The profile of cytokine responses induced *in vitro* by new PRRSV strains detected on farm could define a risk of serious clinical outcomes of PRRSV infection [30,56]; such “immunotypes” might be more important than the usual variants revealed by sequencing of ORF 5 and ORF7 genes. The IgA response to PRRSV in OF, as a possible marker of effective acclimatization of gilts and sows [86], might also be of some importance.

The above approaches to disease control are summarized in Table 1.

## 15. Conclusions

The above issues demand evidence-based responses from the scientific community. It goes without saying that such issues pertain to different areas of research and practitioner activities, focusing on improved disease control actions and surveillance. We need multi-disciplinary contributions to the fields of pig farming, clinical sciences, husbandry, genetics, immunology, and virology, with very clear translational perspectives. These contributions should hopefully generate developments in each of the above translational areas.

## Figures and Tables

**Table 1:** Major issues underlying a more effective control of PRRS.

	Possible Aims	Constraints
**Farm management**	Biosafety. Reduction of environmental infectious pressure.	Lack of adequate facilities, type of farm (farrow-to finish), lack of validated markers.
**Genetic selection**	PRRSV-resistant pigs.	Limited knowledge of molecular basis, costs, unfavorable pig phenotypes (lean type).
**Animal Welfare**	Prevention of stress-related immunosuppression.	Poor housing and infrastructure, high animal densities, unfavorable pig phenotypes.
**Immune control**	Adequate innate and adaptive immune responses by means of vaccines and immunomodulators.	Effective PRRSV decoy strategies, poor recognition of “danger”.
**Markers of risk**	Early warning of possibly serious clinical outcomes and pathogenicity of PRRSV isolates.	Lack of large-scale validations, costs, lack of recognized sampling protocols and laboratory procedures.

## References

[1] Holtkamp D.J. (2013). Assessment of the economic impact of porcine reproductive and respiratory syndrome virus on United States pork producers. J. Swine Health Prod..

[2] Zimmerman J., Benfield D.A., Murtaugh M.P., Osorio F., Stevenson G.W., Tottemorell M. (2006). Porcine reproductive and Respiratory Syndrome Virus (Porcine Arterivirus). Dis. Swine.

[3] Crisci E., Fraile L., Montoya M. (2019). Cellular Innate Immunity against PRRSV and Swine Influenza Viruses. Vet. Sci..

[4] Amadori M., Razzuoli E. (2014). Immune Control of PRRS: Lessons to be Learned and Possible Ways Forward. Front. Vet. Sci..

[5] Bøtner A. (1997). Diagnosis of PRRS. Vet. Microbiol..

[6] Turlewicz-Podbielska H., Włodarek J., Pomorska-Mól M. (2020). Noninvasive strategies for surveillance of swine viral diseases: A review. J. Vet. Diagn Investig..

[7] Corzo C.A., Mondaca E., Wayne S., Torremorell M., Dee S., Davies P., Morrison R.B. (2010). Control and elimination of porcine reproductive and respiratory syndrome virus. Virus Res..

[8] Holtkamp D.J., Polson D.D., Torremorell M., Morrison B., Classen D.M., Becton L., Henry S., Rodibaugh M.T., Rowland R.R., Snelson H. (2011). Terminology for classifying swine herds by porcine reproductive and respiratory syndrome virus status. Tierarztl Prax. Ausg. G. Grosstiere Nutztiere.

[9] Klinge K.L., Vaughn E.M., Roof M.B., Bautista E.M., Murtaugh M.P. (2009). Age-dependent resistance to Porcine reproductive and respiratory syndrome virus replication in swine. Virol. J..

[10] Vashisht K., Erlandson K.R., Firkins L.D., Zuckermann F.A., Goldberg T.L. (2008). Evaluation of contact exposure as a method for acclimatizing growing pigs to porcine reproductive and respiratory syndrome virus. J. Am. Vet. Med. Assoc..

[11] Singleton H., Graham S.P., Bodman-Smith K.B., Frossard J.P., Steinbach F. (2016). Establishing Porcine Monocyte-Derived Macrophage and Dendritic Cell Systems for Studying the Interaction with PRRSV-1. Front. Microbiol..

[12] Bordon Y. (2014). Macrophages: Innate memory training. Nat. Rev. Immunol..

[13] Lewis C.R., Ait-Ali T., Clapperton M., Archibald A.L., Bishop S. (2007). Genetic perspectives on host responses to porcine reproductive and respiratory syndrome (PRRS). Viral Immunol..

[14] Brambilla G., Civitareale C., Ballerini A., Fiori M., Amadori M., Archetti L.I., Regini M., Betti M. (2002). Response to oxidative stress as a welfare parameter in swine. Redox Rep..

[15] van Gucht S., van Reeth K., Pensaert M. (2003). Interaction between porcine reproductive-respiratory syndrome virus and bacterial endotoxin in the lungs of pigs: Potentiation of cytokine production and respiratory disease. J. Clin. Microbiol..

[16] Zhiping W., Malmberg P., Larsson B.M., Larsson K., Larsson L., Saraf A. (1996). Exposure to bacteria in swine-house dust and acute inflammatory reactions in humans. Am. J. Respir. Crit. Care Med..

[17] Boddicker N., Waide E.H., Rowland R.R., Lunney J.K., Garrick D.J., Reecy J.M., Dekkers J.C. (2012). Evidence for a major QTL associated with host response to porcine reproductive and respiratory syndrome virus challenge. J. Anim. Sci..

[18] Burkard C., Opriessnig T., Mileham A.J., Stadejek T., Ait-Ali T., Lillico S.G., Whitelaw C.B.A., Archibald A.L. (2018). Pigs Lacking the Scavenger Receptor Cysteine-Rich Domain 5 of CD163 Are Resistant to Porcine Reproductive and Respiratory Syndrome Virus 1 Infection. J. Virol..

[19] Broom D.M. (1991). Animal welfare: Concepts and measurement. J. Anim. Sci..

[20] Lunney J.K., Fang Y., Ladinig A., Chen N., Li Y., Rowland B., Renukaradhya G.J. (2016). Porcine Reproductive and Respiratory Syndrome Virus (PRRSV): Pathogenesis and Interaction with the Immune System. Annu. Rev. Anim. Biosci..

[21] Mateu E., Diaz I. (2008). The challenge of PRRS immunology. Vet. J..

[22] Kimman T.G., Cornelissen L.A., Moormann R.J., Rebel J.M., Stockhofe-Zurwieden N. (2009). Challenges for porcine reproductive and respiratory syndrome virus (PRRSV) vaccinology. Vaccine.

[23] Yoo D., Song C., Sun Y., Du Y., Kim O., Liu H.C. (2010). Modulation of host cell responses and evasion strategies for porcine reproductive and respiratory syndrome virus. Virus Res..

[24] Costers S., Delputte P.L., Nauwynck H.J. (2006). Porcine reproductive and respiratory syndrome virus-infected alveolar macrophages contain no detectable levels of viral proteins in their plasma membrane and are protected against antibody-dependent, complement-mediated cell lysis. J. Gen. Virol..

[25] Costers S., Lefebvre D.J., Goddeeris B., Delputte P.L., Nauwynck H.J. (2009). Functional impairment of PRRSV-specific peripheral CD3+CD8high cells. Vet. Res..

[26] Murtaugh M.P., Xiao Z., Zuckermann F. (2002). Immunological responses of swine to porcine reproductive and respiratory syndrome virus infection. Viral Immunol..

[27] Dotti S., Villa R., Sossi E., Guadagnini G., Salvini F., Ferrari M., Amadori M. (2011). Comparative evaluation of PRRS virus infection in vaccinated and naive pigs. Res. Vet. Sci..

[28] Nodelijk G., de Jong M.C., van Leengoed L.A., Wensvoort G., Pol J.M., Steverink P.J., Verheijden J.H. (2001). A quantitative assessment of the effectiveness of PRRSV vaccination in pigs under experimental conditions. Vaccine.

[29] Dotti S., Guadagnini G., Salvini F., Razzuoli E., Ferrari M., Alborali G.L., Amadori M. (2013). Time-course of antibody and cell-mediated immune responses to Porcine Reproductive and Respiratory Syndrome virus under field conditions. Res. Vet. Sci..

[30] Ferlazzo G., Ruggeri J., Boniotti M.B., Guarneri F., Barbieri I., Tonni M., Bertasio C., Alborali G.L., Amadori M. (2020). In vitro Cytokine Responses to Virulent PRRS Virus Strains. Front. Vet. Sci..

[31] Morgan S.B., Graham S.P., Salguero F.J., Sanchez Cordon P.J., Mokhtar H., Rebel J.M., Weesendorp E., Bodman-Smith K.B., Steinbach F., Frossard J.P. (2013). Increased pathogenicity of European porcine reproductive and respiratory syndrome virus is associated with enhanced adaptive responses and viral clearance. Vet. Microbiol..

[32] Lunney J.K., Fritz E.R., Reecy J.M., Kuhar D., Prucnal E., Molina R., Christopher-Hennings J., Zimmerman J., Rowland R.R. (2010). Interleukin-8, interleukin-1beta, and interferon-gamma levels are linked to PRRS virus clearance. Viral Immunol..

[33] Li L., Wei Z., Zhou Y., Gao F., Jiang Y., Yu L., Zheng H., Tong W., Yang S., Zheng H. (2015). Host miR-26a suppresses replication of porcine reproductive and respiratory syndrome virus by upregulating type I interferons. Virus Res..

[34] Chen J., Shi X., Zhang X., Wang A., Wang L., Yang Y., Deng R., Zhang G.P. (2017). MicroRNA 373 Facilitates the Replication of Porcine Reproductive and Respiratory Syndrome Virus by Its Negative Regulation of Type I Interferon Induction. J. Virol..

[35] Chang X., Shi X., Zhang X., Chen J., Fan X., Yang Y., Wang L., Wang A., Deng R., Zhou E. (2020). miR-382-5p promotes porcine reproductive and respiratory syndrome virus (PRRSV) replication by negatively regulating the induction of type I interferon. FASEB J..

[36] Prieto C., Alvarez E., Martinez-Lobo F.J., Simarro I., Castro J.M. (2008). Similarity of European porcine reproductive and respiratory syndrome virus strains to vaccine strain is not necessarily predictive of the degree of protective immunity conferred. Vet. J..

[37] Zuckermann F.A., Garcia E.A., Luque I.D., Christopher-Hennings J., Doster A., Brito M., Osorio F. (2007). Assessment of the efficacy of commercial porcine reproductive and respiratory syndrome virus (PRRSV) vaccines based on measurement of serologic response, frequency of gamma-IFN-producing cells and virological parameters of protection upon challenge. Vet. Microbiol..

[38] Diaz I., Ganges L., Galindo-Cardiel I., Tarradas J., Alvarez B., Lorca-Oro C., Pujols J., Gimeno M., Darwich L., Domingo M. (2013). Immunization with DNA vaccines containing porcine reproductive and respiratory syndrome virus open reading frames 5, 6, and 7 may be related to the exacerbation of clinical disease after an experimental challenge. Viral Immunol..

[39] Diaz I., Gimeno M., Darwich L., Navarro N., Kuzemtseva L., Lopez S., Galindo I., Segales J., Martin M., Pujols J. (2012). Characterization of homologous and heterologous adaptive immune responses in porcine reproductive and respiratory syndrome virus infection. Vet. Res..

[40] Park C., Seo H.W., Han K., Kang I., Chae C. (2014). Evaluation of the efficacy of a new modified live porcine reproductive and respiratory syndrome virus (PRRSV) vaccine (Fostera PRRS) against heterologous PRRSV challenge. Vet. Microbiol..

[41] Do D., Nguyen T., Nguyen N., Nguyen M., Le H., Nguyen N., Nguyen N., Chae C., Mah C. (2020). The efficacy and performance impact of Fostera PRRS in a Vietnamese commercial pig farm naturally challenged by a highly pathogenic PRRS virus. Trop. Anim. Health Prod..

[42] Dwivedi V., Manickam C., Patterson R., Dodson K., Murtaugh M., Torrelles J.B., Schlesinger L.S., Renukaradhya G.J. (2011). Cross-protective immunity to porcine reproductive and respiratory syndrome virus by intranasal delivery of a live virus vaccine with a potent adjuvant. Vaccine.

[43] Dwivedi V., Manickam C., Binjawadagi B., Renukaradhya G.J. (2013). PLGA nanoparticle entrapped killed porcine reproductive and respiratory syndrome virus vaccine helps in viral clearance in pigs. Vet. Microbiol..

[44] Ladinig A., Ashley C., Detmer S.E., Wilkinson J.M., Lunney J.K., Plastow G., Harding J.C. (2015). Maternal and fetal predictors of fetal viral load and death in third trimester, type 2 porcine reproductive and respiratory syndrome virus infected pregnant gilts. Vet. Res..

[45] Razzuoli E. Early immune responses to infection by attenuated and non-attenuated, type I PRRS virus strains. Proceedings of the 4th European Veterinary Immunology Workshop.

[46] Davidson S., Maini M.K., Wack A. (2015). Disease-promoting effects of type I interferons in viral, bacterial, and coinfections. J. Interferon Cytokine Res..

[47] Summerfield A., Alves M., Ruggli N., de Bruin M.G., McCullough K.C. (2006). High IFN-alpha responses associated with depletion of lymphocytes and natural IFN-producing cells during classical swine fever. J. Interferon Cytokine Res..

[48] Franzoni G., Edwards J.C., Kurkure N.V., Edgar D.S., Sanchez-Cordon P.J., Haines F.J., Salguero F.J., Everett H.E., Bodman-Smith K.B., Crooke H.R. (2014). Partial Activation of natural killer and gammadelta T cells by classical swine fever viruses is associated with type I interferon elicited from plasmacytoid dendritic cells. Clin. Vaccine Immunol..

[49] Razzuoli E., Villa R., Ferrari A., Amadori M. (2014). A pig tonsil cell culture model for evaluating oral, low-dose IFN-alpha treatments. Vet. Immunol. Immunopathol..

[50] Candotti P., Dotti S., Guana S., Rota Nodari S., Amadori M., Villa R., Petrini S., Lombardi G., Ferrari M. Susceptibility of pure bred Large White and Landrace pigs to experimental infection with porcine reproductive and respiratory syndrome virus. Proceedings of the 20th IPVS Congress (PRRSV).

[51] Lauw F.N., Pajkrt D., Hack C.E., Kurimoto M., van Deventer S.J., van der Poll T. (2000). Proinflammatory effects of IL-10 during human endotoxemia. J. Immunol..

[52] Tilg H., van Montfrans C., van den Ende A., Kaser A., van Deventer S.J., Schreiber S., Gregor M., Ludwiczek O., Rutgeerts P., Gasche C. (2002). Treatment of Crohn’s disease with recombinant human interleukin 10 induces the proinflammatory cytokine interferon gamma. Gut.

[53] Summerfield A., Guzylack-Piriou L., Schaub A., Carrasco C.P., Tache V., Charley B., McCullough K.C. (2003). Porcine peripheral blood dendritic cells and natural interferon-producing cells. Immunology.

[54] Baumann A., Mateu E., Murtaugh M.P., Summerfield A. (2013). Impact of genotype 1 and 2 of porcine reproductive and respiratory syndrome viruses on interferon-alpha responses by plasmacytoid dendritic cells. Vet. Res..

[55] Aman M.J., Tretter T., Eisenbeis I., Bug G., Decker T., Aulitzky W.E., Tilg H., Huber C., Peschel C. (1996). Interferon-alpha stimulates production of interleukin-10 in activated CD4+ T cells and monocytes. Blood.

[56] Gimeno M., Darwich L., Diaz I., de la Torre E., Pujols J., Martin M., Inumaru S., Cano E., Domingo M., Montoya M. (2011). Cytokine profiles and phenotype regulation of antigen presenting cells by genotype-I porcine reproductive and respiratory syndrome virus isolates. Vet. Res..

[57] Levings M.K., Sangregorio R., Galbiati F., Squadrone S., de Waal Malefyt R., Roncarolo M.G. (2001). IFN-alpha and IL-10 induce the differentiation of human type 1 T regulatory cells. J. Immunol..

[58] Silva-Campa E., Flores-Mendoza L., Resendiz M., Pinelli-Saavedra A., Mata-Haro V., Mwangi W., Hernandez J. (2009). Induction of T helper 3 regulatory cells by dendritic cells infected with porcine reproductive and respiratory syndrome virus. Virology.

[59] Wang J., Gong L., Zhang W., Chen W., Pan H., Zeng Y., Liang X., Ma J., Zhang G., Wang H. (2020). Wnt/?-catenin signaling pathway inhibits porcine reproductive and respiratory syndrome virus replication by enhancing the nuclear factor-?B-dependent innate immune response. Vet. Microbiol..

[60] Miller L.C., Fleming D.S., Li X., Bayles D.O., Blecha F., Sang Y. (2017). Comparative analysis of signature genes in PRRSV-infected porcine monocyte-derived cells to different stimuli. PLoS ONE.

[61] Balasuriya U.B., MacLachlan N.J. (2004). The immune response to equine arteritis virus: Potential lessons for other arteriviruses. Vet. Immunol. Immunopathol..

[62] Rowland R.R., Even C., Anderson G.W., Chen Z., Hu B., Plagemann P.G. (1994). Neonatal infection of mice with lactate dehydrogenase-elevating virus results in suppression of humoral antiviral immune response but does not alter the course of viraemia or the polyclonal activation of B cells and immune complex formation. J. Gen. Virol..

[63] Balasuriya U.B., Carossino M. (2017). Reproductive effects of arteriviruses: Equine arteritis virus and porcine reproductive and respiratory syndrome virus infections. Curr. Opin. Virol..

[64] Snijder E.J., Kikkert M., Fang Y. (2013). Arterivirus molecular biology and pathogenesis. J. Gen. Virol..

[65] Young J.E., Dvorak C.M.T., Graham S.P., Murtaugh M.P. (2021). Isolation of Porcine Reproductive and Respiratory Syndrome Virus GP5-Specific, Neutralizing Monoclonal Antibodies from Hyperimmune Sows. Front. Immunol..

[66] Ostrowski M., Galeota J.A., Jar A.M., Platt K.B., Osorio F.A., Lopez O.J. (2002). Identification of neutralizing and nonneutralizing epitopes in the porcine reproductive and respiratory syndrome virus GP5 ectodomain. J. Virol..

[67] Wissink E.H.J., van Wijk H.A.R., Kroese M.V., Weiland E., Meulenberg J.J.M., Rottier P.J.M., van Rijn P.A. (2003). The major envelope protein, GP5, of a European porcine reproductive and respiratory syndrome virus contains a neutralization epitope in its N-terminal ectodomain. J. Gen. Virol..

[68] Vanhee M., Van Breedam W., Costers S., Geldhof M., Noppe Y., Nauwynck H. (2011). Characterization of antigenic regions in the porcine reproductive and respiratory syndrome virus by the use of peptide-specific serum antibodies. Vaccine.

[69] Su J., Zhou L., He B., Zhang X., Ge X., Han J., Guo X., Yang H. (2019). Nsp2 and GP5-M of Porcine Reproductive and Respiratory Syndrome Virus Contribute to Targets for Neutralizing Antibodies. Virol. Sin..

[70] Goldeck D., Perry D.M., Hayes J.W.P., Johnson L.P.M., Young J.E., Roychoudhury P., McLuskey E.L., Moffat K., Bakker A.Q., Kwakkenbos M.J. (2019). Establishment of Systems to Enable Isolation of Porcine Monoclonal Antibodies Broadly Neutralizing the Porcine Reproductive and Respiratory Syndrome Virus. Front. Immunol..

[71] Schlake T., Thran M., Fiedler K., Heidenreich R., Petsch B., Fotin-Mleczek M. (2019). mRNA: A Novel Avenue to Antibody Therapy?. Mol. Ther..

[72] Thess A., Grund S., Mui B.L., Hope M.J., Baumhof P., Fotin-Mleczek M., Schlake T. (2015). Sequence-engineered mRNA without Chemical Nucleoside Modifications Enables an Effective Protein Therapy in Large Animals. Mol. Ther..

[73] Carlsson L., Clarke J.C., Yen C., Gregoire F., Albery T., Billger M., Egnell A.C., Gan L.M., Jennbacken K., Johansson E. (2018). Biocompatible, Purified VEGF-A mRNA Improves Cardiac Function after Intracardiac Injection 1 Week Post-myocardial Infarction in Swine. Mol. Ther. Methods Clin. Dev..

[74] Pardi N., Secreto A.J., Shan X., Debonera F., Glover J., Yi Y., Muramatsu H., Ni H., Mui B.L., Tam Y.K. (2017). Administration of nucleoside-modified mRNA encoding broadly neutralizing antibody protects humanized mice from HIV-1 challenge. Nat. Commun..

[75] Thran M., Mukherjee J., Ponisch M., Fiedler K., Thess A., Mui B.L., Hope M.J., Tam Y.K., Horscroft N., Heidenreich R. (2017). mRNA mediates passive vaccination against infectious agents, toxins, and tumors. EMBO Mol. Med..

[76] Osorio F.A., Galeota J.A., Nelson E., Brodersen B., Doster A., Wills R., Zuckermann F., Laegreid W.W. (2002). Passive transfer of virus-specific antibodies confers protection against reproductive failure induced by a virulent strain of porcine reproductive and respiratory syndrome virus and establishes sterilizing immunity. Virology.

[77] Lopez O.J., Oliveira M.F., Garcia E.A., Kwon B.J., Doster A., Osorio F.A. (2007). Protection against porcine reproductive and respiratory syndrome virus (PRRSV) infection through passive transfer of PRRSV-neutralizing antibodies is dose dependent. Clin. Vaccine Immunol..

[78] Robinson S.R., Rahe M.C., Gray D.K., Martins K.V., Murtaugh M.P. (2018). Porcine reproductive and respiratory syndrome virus neutralizing antibodies provide in vivo cross-protection to PRRSV1 and PRRSV2 viral challenge. Virus Res..

[79] Yoon K.J., Wu L.L., Zimmerman J.J., Platt K.B. (1997). Field isolates of porcine reproductive and respiratory syndrome virus (PRRSV) vary in their susceptibility to antibody dependent enhancement (ADE) of infection. Vet. Microbiol..

[80] Carlson J., O’Donnell V., Alfano M., Velazquez Salinas L., Holinka L.G., Krug P.W., Gladue D.P., Higgs S., Borca M.V. (2016). Association of the Host Immune Response with Protection Using a Live Attenuated African Swine Fever Virus Model. Viruses.

[81] Schlafer D.H., McVicar J.W., Mebus C.A. (1984). African swine fever convalescent sows: Subsequent pregnancy and the effect of colostral antibody on challenge inoculation of their pigs. Am. J. Vet. Res..

[82] Salmon H., Berri M., Gerdts V., Meurens F. (2009). Humoral and cellular factors of maternal immunity in swine. Dev. Comp. Immunol..

[83] Mkaddem S.B., Christou I., Rossato E., Berthelot L., Lehuen A., Monteiro R.C. (2014). IgA, IgA receptors, and their anti-inflammatory properties. Curr. Top. Microbiol. Immunol..

[84] Saha C., Das M., Patil V., Stephen-Victor E., Sharma M., Wymann S., Jordi M., Vonarburg C., Kaveri S.V., Bayry J. (2017). Monomeric Immunoglobulin A from Plasma Inhibits Human Th17 Responses In Vitro Independent of FcalphaRI and DC-SIGN. Front. Immunol..

[85] Maddur M.S., Vani J., Hegde P., Lacroix-Desmazes S., Kaveri S.V., Bayry J. (2011). Inhibition of differentiation, amplification, and function of human TH17 cells by intravenous immunoglobulin. J. Allergy Clin. Immunol..

[86] Drigo M., Giacomini E., Lazzaro M., Pasotto D., Bilato D., Ruggeri J., Boniotti M.B., Alborali G.L., Amadori M. (2018). Comparative evaluation of immune responses of swine in PRRS-stable and unstable herds. Vet. Immunol. Immunopathol..

[87] Ruggeri J., Ferlazzo G., Boniotti M.B., Capucci L., Guarneri F., Barbieri I., Alborali G.L., Amadori M. (2020). Characterization of the IgA response to PRRS virus in pig oral fluids. PLoS ONE.

[88] Mazanec M.B., Kaetzel C.S., Lamm M.E., Fletcher D., Nedrud J.G. (1992). Intracellular neutralization of virus by immunoglobulin A antibodies. Proc. Natl. Acad. Sci. USA.

[89] Nazki S., Khatun A., Jeong C.G., Mattoo S.U.S., Gu S., Lee S.I., Kim S.C., Park J.H., Yang M.S., Kim B. (2020). Evaluation of local and systemic immune responses in pigs experimentally challenged with porcine reproductive and respiratory syndrome virus. Vet. Res..

[90] Xiao Z., Batista L., Dee S., Halbur P., Murtaugh M.P. (2004). The level of virus-specific T-cell and macrophage recruitment in porcine reproductive and respiratory syndrome virus infection in pigs is independent of virus load. J. Virol..

[91] Lohse L., Nielsen J., Eriksen L. (2004). Temporary CD8+ T-cell depletion in pigs does not exacerbate infection with porcine reproductive and respiratory syndrome virus (PRRSV). Viral Immunol..

[92] Darwich L., Diaz I., Mateu E. (2010). Certainties, doubts and hypotheses in porcine reproductive and respiratory syndrome virus immunobiology. Virus Res..

[93] Meier W.A., Galeota J., Osorio F.A., Husmann R.J., Schnitzlein W.M., Zuckermann F.A. (2003). Gradual development of the interferon-gamma response of swine to porcine reproductive and respiratory syndrome virus infection or vaccination. Virology.

[94] Lowe J.E., Husmann R., Firkins L.D., Zuckermann F.A., Goldberg T.L. (2005). Correlation of cell-mediated immunity against porcine reproductive and respiratory syndrome virus with protection against reproductive failure in sows during outbreaks of porcine reproductive and respiratory syndrome in commercial herds. J. Am. Vet. Med. Assoc..

[95] Amadori M., Zanotti C. (2016). Immunoprophylaxis in intensive farming systems: The way forward. Vet. Immunol. Immunopathol..

[96] Gonzàlez S., Lòpez-Larrea C., Lòpez-Soto A., Amadori M. (2016). The Molecular Basis of the Immune Response to Stressed Cells and Tissues. The Innate Immune Response to Noninfectious Stressors.

[97] Welch S.K., Calvert J.G. (2010). A brief review of CD163 and its role in PRRSV infection. Virus Res..

[98] Wesley R.D., Lager K.M., Kehrli M.E. (2006). Infection with Porcine reproductive and respiratory syndrome virus stimulates an early gamma interferon response in the serum of pigs. Can. J. Vet. Res..

[99] Karniychuk U.U., Nauwynck H.J. (2013). Pathogenesis and prevention of placental and transplacental porcine reproductive and respiratory syndrome virus infection. Vet. Res..

[100] Montaner-Tarbes S., Del Portillo H.A., Montoya M., Fraile L. (2019). Key Gaps in the Knowledge of the Porcine Respiratory Reproductive Syndrome Virus (PRRSV). Front. Vet. Sci..

[101] Roca M., Gimeno M., Bruguera S., Segales J., Diaz I., Galindo-Cardiel I.J., Martinez E., Darwich L., Fang Y., Maldonado J. (2012). Effects of challenge with a virulent genotype II strain of porcine reproductive and respiratory syndrome virus on piglets vaccinated with an attenuated genotype I strain vaccine. Vet. J..

[102] Pahl J.H.W., Cerwenka A., Ni J. (2018). Memory-Like NK Cells: Remembering a Previous Activation by Cytokines and NK Cell Receptors. Front. Immunol..

[103] Lopez Fuertes L., Domenech N., Alvarez B., Ezquerra A., Dominguez J., Castro J.M., Alonso F. (1999). Analysis of cellular immune response in pigs recovered from porcine respiratory and reproductive syndrome infection. Virus Res..

[104] Sang Y., Rowland R.R., Blecha F. (2014). Antiviral regulation in porcine monocytic cells at different activation states. J. Virol..

[105] Reddy R.C. (2008). Immunomodulatory role of PPAR-gamma in alveolar macrophages. J. Investig. Med..

[106] Rosas-Ballina M., Guan X.L., Schmidt A., Bumann D. (2020). Classical Activation of Macrophages Leads to Lipid Droplet Formation Without de novo Fatty Acid Synthesis. Front. Immunol..

[107] Zhang K., Hou Q., Zhong Z., Li X., Chen H., Li W., Wen J., Wang L., Liu W., Zhong F. (2013). Porcine reproductive and respiratory syndrome virus activates inflammasomes of porcine alveolar macrophages via its small envelope protein E. Virology.

[108] Wang C., Shi X., Zhang X., Wang A., Wang L., Chen J., Deng R., Zhang G. (2015). The Endoribonuclease Activity Essential for the Nonstructural Protein 11 of Porcine Reproductive and Respiratory Syndrome Virus to Inhibit NLRP3 Inflammasome-Mediated IL-1beta Induction. DNA Cell Biol..

[109] Yu L.X., Wang X., Yu H., Jiang Y.F., Gao F., Tong W., Li L.W., Li H.C., Yang S., Chen P.F. (2018). The emergence of a highly pathogenic porcine reproductive and respiratory syndrome virus with additional 120aa deletion in Nsp2 region in Jiangxi, China. Transbound. Emerg. Dis..

[110] Kittawornrat A., Engle M., Panyasing Y., Olsen C., Schwartz K., Rice A., Lizano S., Wang C., Zimmerman J. (2013). Kinetics of the porcine reproductive and respiratory syndrome virus (PRRSV) humoral immune response in swine serum and oral fluids collected from individual boars. BMC Vet. Res..

[111] Frazzini S., Riva F., Amadori M. (2021). Therapeutic and Prophylactic Use of Oral, Low-Dose IFNs in Species of Veterinary Interest: Back to the Future. Vet. Sci..

